# Navigating the impact of workplace distractions for persons with TBI: a qualitative descriptive study

**DOI:** 10.1038/s41598-022-20083-0

**Published:** 2022-09-23

**Authors:** DeAnna Pinnow, Renee Causey-Upton, Peter Meulenbroek

**Affiliations:** 1 Department of Communication Sciences and Disorders, University of Kentucky, Lexington, KY USA; 2 Department of Occupational Science and Occupational Therapy, Eastern Kentucky University, Richmond, KY USA

**Keywords:** Attention, Cognitive control, Perception, Long-term memory, Short-term memory, Working memory, Human behaviour

## Abstract

Persons with traumatic brain injuries (TBIs) who return to work often struggle with managing environmental distractions due to residual cognitive impairments. Previous literature has established that environmental distractions impact persons with TBI, yet, the extent to which distractions impact workplace performance is unknown. This qualitative descriptive study using phenomenology methods, explored the experiences of seven individuals with TBIs and how they perceived workplace distractions to impact their productivity. Data was collected using semi-structured interviews with seven participants who were diagnosed with mild, moderate, and severe TBIs. Interviews were transcribed and analyzed using thematic analysis. Main findings centered around what environmental distractions impacted work performance, the farther-reaching consequences of distractibility, strong emotional feelings and worry about perceived work performance associated with distractibility, mitigating distractibility through “gaming the attentional system”, and utilizing music as a distraction masker to enhance task performance. In light of this study’s findings, researchers, and clinicians are encouraged to consider the wider impact of distractions on persons with TBI. The real-life accounts documented in this study will assist researchers and clinicians to account for the impact of environmental distractions in rehabilitation and support employment for persons with TBI.

## Introduction

Less than 40% of persons with traumatic brain injuries (TBIs) return to work (RTW) despite rehabilitative efforts to reduce injury-related deficits^1^. These outcomes account for the staggering economic cost associated with TBI which is estimated at 76.5 billion dollars per year^2–4^. Additionally, delayed or a lack of RTW reduces opportunities for persons with TBI to re-build their sense of identity, community integration, family participation, social supports, emotional well-being, and financial stability^5–7^. It has been established that RTW outcomes are predicted by several factors including injury severity, pre-morbid employment status, age, education, and cause of injury^8–10^. However, cognitive, physical, emotional, and behavioral deficits have also been identified as predictors of successful RTW^3^. These factors are modifiable and are prominent goals of rehabilitation^11,12^.

Cognitive impairments have been identified as the most prevalent limitation associated with RTW and TBI^13–15^. In a previous meta-analysis, 18% of persons with mild TBI and 50% of persons with moderate to severe TBI suffer from chronic cognitive impairments (over 1.75 million cases annually), specifically with attention and memory, that are exacerbated by environmental factors such as distractions^16,17^. Distractions can be defined as irrelevant sensory stimuli of any modality (i.e., auditory, visual, audiovisual, tactile, or internal) that negatively impact task completion^18^. After a TBI, the attentional selection process (i.e., selective attention) of relevant and irrelevant (i.e., distractions) sensory stimuli can be impaired. As a result, persons with TBI may operate on irrelevant sensory inputs which cause delays, errors, or multiple attempts in task completion^19–21^. Therefore, distractions have been deemed a barrier to successful RTW leading to the common recommendation to minimize or remove distractions for tasks that require focus^20^. However, in recent years some studies have demonstrated how some distractions can have a positive impact on performance by recruiting additional attentional resources to increase focus^22,23^. Therefore, authors defined distraction as any sensory stimulus that disrupts the encoding, storage, or retrieval for task completion or any sensory stimuli that enhances task performance by increasing attentional focus and decreasing internal factors (i.e., stress, mind wandering)^18^.

Targeting workplace distractibility for persons with TBI continues to be a challenge due to the current ecological validity that threatens the sensitivity of clinical assessments^24,25^. Rehabilitation professionals are limited in their ability to assess breakdowns in cognition due to distractions and adjust treatment plans within clinic-based settings. Therefore, they are reliant on accurate patient-reported breakdowns in cognition in everyday environments which is problematic for persons with TBI who struggle with memory, insight, and awareness^26^. However, a promising solution is the use of computer-assisted programs to simulate everyday environments for cognitive assessment^27^. There are a limited number of studies targeting workplace performance for persons with TBI and even less that include distractions^28–30^. The continued challenge of including distractions in computer-assisted programs is due to the complex relationship between the context of tasks and distraction characteristics (intensity, duration, modality, and location). Unfortunately, distractions included within these programs are pre-determined by researchers instead of by clinical populations^18^. Therefore, qualitative data is required prior to the development of simulation programs in order to customize distractions to elicit desired behavioral outcomes for specific clinical populations. Although studies have endorsed the impact of distractibility in successful RTW for persons with TBI, the extent to which distractions impact work performance is unknown^31^. To identify how functional performance is impaired after TBI with validity, we require rich descriptions of the experiences of persons with TBI and distractions in the workplace. Additionally, participants’ descriptions will help assist in the development of customized simulation programs to assess and train skills for returning to work with environmental distractions. The purpose of this qualitative descriptive study was to describe and compare the common experiences of individuals with TBIs and how they perceived workplace distractions to impact their productivity.

## Methods

Ethical approval was provided by the University of Kentucky institutional review board and was planned and conducted in accordance with the Declaration of Helsinki. Informal assessment of capacity for consent was completed through the University of California, San Diego Brief Assessment of Capacity to Consent (UBACC) by all participants prior to completing the informed consent^32^. A qualitative descriptive study design using phenomenology methods was used to describe the experiences of persons with TBI and how they perceived distractions to impact their work performance^33^. Telephone interviews were conducted to ensure feasibility due to the current employment status of the participants and to follow socially distant guidelines due to the coronavirus (COVID-19).

### Recruitment and participants

Purposeful and snowball recruitment strategies were utilized to identify participants with mild, moderate, or severe TBIs^34^. The Social Communication and Cognitive Abilities (SCCA) laboratory at the University of Kentucky provided a list of previous research participants who were employed and agreed to be contacted for future studies. Recruitment was also done in collaboration with the University of Kentucky Center for Clinical and Translation Science (CCTS) for advertising purposes. Individuals were contacted with a recruitment phone call and email that included information about the study and the primary investigator’s contact information. Participants were included if they had either employment or volunteer experience in the previous six months, diagnosed with a TBI, and spoke English. Recruitment continued until data saturation was reached, meaning no new information emerged from data obtained from later participants. Data saturation was met after participant five, however, two additional participants were interviewed to confirm study themes. All participants who expressed interest in the study participated.

### Data collection

All data was collected remotely during the COVID-19 global pandemic. Authors obtained baseline information regarding TBI severity level, employment status pre and post injury, skill level, and Cognitive Failures Questionnaire (CFQ) scores. The CFQ is a 25-item questionnaire that assesses the frequency of cognitive failures that included absent-mindedness, errors of perception, memory, and motor functioning^35^. Scores are meant to be predictive of situations of absent-mindedness in both a laboratory setting and everyday life. The CFQ score is a summation of all answers in which scores can range from 0 to 100 with a higher score indicating cognitive difficulties. All participants completed the CFQ online prior to the interview to minimize the potential fatigue. Participants also completed one on one semi-structured telephone interviews with the primary investigator to ensure consistency in interview techniques. Open ended questions were utilized to learn more about participants subjective experiences and to provide them with the opportunity to talk freely^33^. The interview guide consisted of 15 questions that asked participants to describe distractions in their work environment and to provide their perceptions about how distractions impacted their work performance (see Supplementary Material). Recorded telephone interviews ranged from 45 to 60 min and were transcribed verbatim.

### Data analysis

Thematic analysis was conducted on interview transcriptions concurrently; the primary author began analysis while collecting data through subsequent interviews until saturation was met. The first author developed initial impressions from the data through reading each transcription in its entirety. Transcriptions were then analyzed for significant statements which were recorded without a pre-designed order using horizontalization to reflect the range of participants’ experiences^36,37^. The primary author and second author reviewed significant statements separately, then compared initial findings to improve reliability and to check for consistency. Significant statements where then labeled and combined into initial themes^33^. Authors then reflected on the significant statements and initial themes to develop the final study themes and subthemes. Final themes and subthemes were presented to the third author and any disagreements were resolved.

### Research team and reflexivity

The first and third authors are speech-language pathologists with clinical expertise in persons with TBI. Additionally, the third author is an associate professor and clinical researcher. The second author is an associate professor, clinical researcher, and occupational therapist expertise in qualitative methodology. Several strategies were implemented to ensure trustworthiness including a reflexivity journal, secondary review of transcriptions by an independent reviewer after the initial transcription, peer review with a qualitative researcher during each step of the development of significant statements to final study themes, and an audit trail. Lastly, due to the possibility of participants’ responses being influenced by external and internal distractions within in individual interview environments (i.e., own homes), interview strategies were implemented to confirm the primary investigator’s understanding of responses from the participants.

### Ethical approval

This study, project number 5895, was approved by the University of Kentucky Institutional Review Board (IRB). It was planned and conducted in accordance with the Declaration of Helsinki.

## Results

Seven participants completed interviews; one male and six females. Participants were diagnosed with mild, moderate, or severe TBI and ranged from 6 months to 40-years post injury. Due to COVID-19, employment status fluctuated for the participants, requiring them to either work from home, adjust work hours, or obtain a new job. Therefore, participants’ employment status ranged from part-time to full-time (Table 1). Employment positions for the participants included unskilled labor requiring little, if any, education and training, semi-skilled labor requiring some education and training, and skilled labor requiring specialized education and training^38,39^. Participants’ CFQ scores ranged from 23 to 93, with lower scores reflecting minimal cognitive difficulties and larger scores reflecting more severe cognitive difficulties**.**

**Table 1:** Participant demographics.

Participant identifier	Self-reported TBI severity level	CFQ score (Out of 100)	Employment status prior to TBI	Current employment status	Part-time definition	Skill level
M1	Mild	24	In school	Full-time	30–40 h per week	Skilled
F2	Severe	49	Employed	Part-time	2–3 days monthly	Semi-skilled
F3	Mild	48	Employed	Part-time	20 h per week	Skilled
F4	Mild	38	Employed	Part-time	20 h per week	Skilled
F5	Mild	32	Employed	Part-time	20 h per week	Semi-skilled
F6	Mild	36	Employed	Part-time	2–3 days monthly	Unskilled
F7	Severe	93	Employed	Full-time	40 h per week	Semi-skilled

CFQ scores are cumulative and can range from 0 to 100 with a higher score indicating subjective cognitive difficulties. A high score is defined as a score ≥ 43.

Despite reported differences in workplace environments, all participants endorsed experiencing distractions in the workplace that impacted their work performance. Although distractibility can be highly individualized, five themes and nine subthemes emerged from the data (Table 2). Themes and subthemes are described in detail in the sections that follow with verbatim quotes from the interview transcripts.

**Table 2:** Master themes and sub-themes.

Master themes	Sub-themes
Environmental distractions impact reliable work performance	Work performance outcomes External distractions (auditory and visual) Internal distractions
Consequences of distractibility reach beyond task outcomes	Social consequences with colleagues Financial consequences
Distractibility creates strong emotional feelings and worry about perceived work performance	Fear and anxiety about others’ perceptions of work performance Chain effect on emotions
Mitigating distractibility requires “gaming the system”	Beating the fatigue clock Environmental supports
Utilizing music as a distraction masker to enhance task performance	

### Theme 1: environmental distractions impact reliable work performance

#### Unreliable work performance was endorsed by every participant as the result of environmental distractions in the workplace

Participants reported that recognizing when they had become distracted was challenging. As a result, they experienced significant delays in task completion, repeated the same task several times, or completed the task with errors. Participant 3 explained: “It’s always reading that, I’ll get through it, and I remember reading the words and paying attention, but I have like a whole other thought going and then like s*** or crap I just finished this page. ‘What did that say?’ I have no idea, I haven’t processed it, and I’ll have to read it four five more times”. Participant 7 shared a similar experience: “I’ll get to work and even in my own house, and I’ll be sitting here doing something and I walk into a room forget what I’ve walked in there for or I’ll be doing something and then see something else like ‘squirrel’ that draws my attention to it and start doing that and completely forget what else I needed to do”.

#### Specific distraction characteristics such as modality (i.e., external, or internal), and intensity (i.e., loudness, or number of distractions present at once) disrupted participants’ work performance

Participants reported a variety of workplace distractions that impacted work performance such as co-workers, customers, loud noises, background chatter, and the content of background chatter. All participants reported that working around either customers or co-workers was necessary for their employment, however they found it challenging to maintain their focus on work tasks. Participant 4 said: “Um I, I hate talking to more than like two or three people at a time, so anytime there’s more than two or three people around me even if they’re not listening into the conversation. I automatically like, I always get distracted by other people being around especially if I don’t know them super well”. Ironically, participants described scenarios in which they became distracted by listening to co-workers’ conversations hoping to learn job-related information while also experiencing delays or errors in task completion at the same time. However, not all information was pertinent to participants’ assigned work tasks. Participant 7 explained: “I’m around a bunch of new stuff, so I’m having to try to learn all that but then you know I try to listen to like what the other employees are saying you know cause it may be something I need to know or learn you know”. In addition to external distractions, participants described how the intensity of distractions impacted their ability to focus on work tasks. Participant 3 said: “Noise, noise if there were if there was a lot of noise and a lot of people talking near me and a lot of people trying to talk to me it’s the noise overload, it’s always the big thing that gets me” and “once it [noise] hits a certain threshold I have a really hard time focusing”.

#### Internal distractions were recognized by all participants as a significant contribution to breakdowns in attention at work

 Participants explained how internal distractions such as stress, anxiety, and thinking about items unrelated to work tasks impacted their productivity. Participants were not always aware they had become internally distracted until a significant amount of time had passed or they could not recall what task they were completing. Participant 4 explained: “I’ll be like, I’ll be you know, I’ll get a few sentences in and be like ‘Okay this is making sense, oh this reminds me I should, like what was I gonna, you know make for dinner later or do I need to do this also?’ ‘Oh yeah I need to like call so and so, I need to eat, respond to that email, oh crap where was that in my reading, oh there wait what was it talking about?’” Participant 6 reported: “Stress usually like drives me to do it [get distracted] because of like anxiety and I probably won’t get it done in time or like stuff like that so like when it [high level of anxiety/stress] is like the biggest”. Interestingly, when participants attempted to remove all distractions, they noticed an influx of internal distractions. Participant 4 stated: “Oh yeah, like yes those [internal distractions] are probably the most distracting things especially when it’s quiet”.

### Theme 2: consequences of distractibility reach beyond task outcomes

#### Distractibility in the workplace resulted in consequences that reached beyond the completion of assigned work tasks

Specifically, participants described experiences with financial and social consequences due to distractibility in addition to errors or delays in task completion. Financial consequences ranged from deductions in pay, suspension without pay, or being fired. Participants reported problems with mental math, such as balancing a cashier till. This resulted in consequences including taking money out of participants’ wages to make up for the accounting errors and even suspension without pay. Participant 2 described: “I’ve gotten written up, I’ve come up short quite often. Well, I’ve come up short when I first started, I was short all the time and I kept telling [the boss], he’s like, ‘God, you know, what are you doing?’ I’m like, ‘Oh, I love this job! I’ll get better, I’ll get better’. Cause I wasn’t careful with my money, and I’ve learned to be careful. And I have gotten written up numerous times and enough times where I got, I couldn’t work for three days, I forget what it’s called, they wouldn’t let me work for 3 days so I missed 3 days of pay”. Formal write-ups from employers were an additional consequence due to distractibility. Participant 7 explained: “Being written up is the biggest thing, I’ve been suspended you know for not stopping properly, you know passing somebody on a stop, and you know because they point all that out you know. Because that’s their job is to pick people up [bus drivers] and you know if you’re distracted and tired because you don’t see somebody then you know they’ll write you up for things”.

#### Participants reported negative social consequences with their colleagues due to distractibility and were often misperceived as rude when they had become distracted

These scenarios occurred when participants’ intentions were to be a good employee by either focusing on their work or helping co-workers. Additionally, they felt torn between interacting with customers, helping their colleagues, and focusing on their work tasks. Participant 2 said: “I can hear people and making bets (gambling) beside me and when the agent (co-worker) doesn’t know what the bet is I will say ‘Come over here because you are doing it wrong’. So that affects me. I got in trouble with one of my coworkers and they got mad at me. I was like ‘You didn’t know what the bet was’ and they were like ‘Well that doesn’t mean you need to interrupt’”. Participant 6 stated: “The people we’re working for like talk to me and stuff, and then I feel rude like not to respond and like stop what I’m doing”.

### Theme 3: distraction creates strong emotional feelings and worry about perceived work performance

#### Participants described a cascading effect of distraction on their emotions which fluctuated from frustration, anger, a sense of let down, and disbelief

Feelings of annoyance and letting themselves or others down due to distractibility was commonly reported. Specifically, participants wanted to be viewed as a valuable employee and co-worker. Therefore, participants’ desired work-identity was jeopardized due to their distractibility, which negatively impacted their emotional control. Participant 3 stated: “It’s really frustrating for me to just sit there and think like, I know I’m better than this, I know I’m capable of this, but like I can’t get my brain to work right. That’s a common problem I have”. Participant 1 shared a similar view: “Like I said, I don’t feel like I really get distracted but when I do I guess a little bit of frustration and a little bit of I don’t know, but disbelief just like frustrated with myself really for like succumbing the distraction”. Additionally, participants required extra time to complete work tasks to manage both their emotions and distractions. Participant 6 explained “When I get distracted it’s more of like getting annoyed with myself ‘cuz like I know I have to get something done, but it’s just like taking a lot longer to do it”.

#### All participants expressed a strong desire to be viewed as competent in the workplace and feared that their distractibility would diminish others’ perception of their capabilities

 Wanting to maintain employment led participants to become fearful of losing their job due to difficulties with managing distractions. Participant 1 said: “I do personally like value being seen as competent. I think that I think values hold such a such a high place in the character we have after brain injury especially because if you don’t care about being seen as competent it like none of this really matters you know I mean”. Participants explained that they were fearful of co-workers or employers noticing their distractibility during work meetings or conversations. Participant 4 explained: “Oh yeah, there’s something else going on right in front of me that I’m supposed to be paying attention to and haven’t been paying attention to. And then I’m like, then I almost have like an anxiety reaction to like, ‘Oh my gosh, what have we been talking about? What if someone calls me? What if they ask me a question and I don’t know what I’m doing?’ type thing”. Being viewed as competent or fit for employment led some participants to conceal their cognitive deficits and TBI diagnosis. Participant 7 explained: “I’m like that. I don’t want people to sit here and think oh you’re stupid you know. You can’t do anything right, you know, especially for the people that don’t know that, oh well, she’s had a brain injury, you know. I don’t go and try to tell everybody I’ve got a brain injury because then you know nobody, nobody is going to hire you for a job”.

### Theme 4: mitigating distractibility requires “gaming the system”

Over time and with repeated exposure to individual work environments participants grew to understand what distractions disrupted their attention and memory. As a result, participants endorsed manipulating factors within themselves (i.e., fatigue) or within their environment to achieve desired work outcomes, or “gaming attentional the system”. Without repeated exposure to workplace distractions in conjunction with work tasks participants would have struggled to identify what strategies worked and which ones did not.

#### Neurofatigue contributed to all participants’ distractibility causing them adjust work hours to beat their internal clock of fatigue

 As participants’ fatigue increased, they noticed subsequent increases in distractibility. Therefore, some participants arranged to adjust their work hours to early mornings to accommodate their fatigue levels as the day progressed. Participant 3 explained: “It depends on how fatigued I am. So, if I’m fatigued, it [distractibility] is usually going to happen later in the day”. Participant 1 said: “Well it’s, well, as I go through my day say around like I’m done usually around, I don’t know, 2:30/3:00 and that’s because then that time of day is when my cognitive fatigue really kicks in. And like that’s when I really would see problems if I was tryin’ to solve problems and like interact with other people. I, I’d get really kind of upset for, not all upset, I just, it just, it’s frustrating cause I know, I know I can do this stuff but I get my brain is not really cooperating with me at that time so I can only think clearly like I should be able to you know I mean?”.

#### Environmental supports and strategies were required for participants to focus their attention throughout the workday

Participants described strategies that varied from working as a team with co-workers, de-cluttering their workspace, reviewing open computer tabs, and fidgeting, to monitoring others’ expressions/non-verbal feedback. Strategies were developed by participants through trial and error during work tasks while distractions were present. Participant 3 noted: “I had a, I had a staff [co-worker] with me and it was helpful”. Participant 2 stated: “I like to de-clutter. I will count my money on my chair so that I get away from everybody, from all other distractions.” Participant 1 explained: “I’ll just pick up on social cues, like I can just, like as if I’d ever do it, I’ll self-talk to myself about stuff like ‘What are you doing [first name]?’ Or I’ll realize someone’s not reacting to something like I think they should be. I think that’s the biggest cue and just social interactions non-verbal communication with people”. Participant 4 explained: “And so I just end up looking at my tabs [computer] usually they are kind of my train of thoughts and then I have to like work backwards”.

### Theme 5: utilizing music as a distraction masker to enhance task performance

#### Six participants endorsed utilizing music as a strategy to minimize internal and external distractions

Surprisingly minimizing all external distractions in the workplace increased participants’ internal distractions. With minimal options left for increasing focus, participants utilized instrumental music, a form of distraction, to mask all other modalities of distraction. Participant 4 explained: “If I’m trying to read something usually end up turning either everything off or turning like a symphony station or classical music station on, or even like a white noise like rainymood.com or something like that”. Participant 3 described a similar experience: “I use music if I don’t have some kind of like white noise in the background. Say, if I’m reading an article or trying to really laser focus in on an eval if it’s like a really difficult one or something I’m not used to. I almost use a white noise to tune everything else out. So, I’ll have music playing in the background as I’m studying, it’s instrumental so there’s not words to distract me, or if in a language I can’t understand. Just things like that in those high-stakes situations”.

## Discussion

This qualitative descriptive study was part of a series of studies investigating the impact of distractions on cognition for persons with TBI and healthy controls. Specifically, the study explored the experiences of seven individuals with TBI and their perceptions of how distractions impacted their work performance. Participants endorsed the impact of distractions on key soft and hard skills that are desired by employers such as social communication, mental flexibility, timeliness, and money management^40,41^. As a result, participants experienced consequences both financially and socially. All participants shared a strong desire to maintain their employment and to be viewed as competent in the workplace but were concerned how their distractibility was viewed by others. The repeated exposure to distractions during work tasks enabled participants to identify strategies to manipulate or “game” their own attentional system for optimizing productivity. Surprisingly, a key strategy for minimizing environmental distractions was the use of instrumental music. This new finding may be indicative that distractions with specific stimulus characteristics can enhance task performance. Therefore, the data presented in this study indicates that distractions can be both task disrupting and task enhancing or ‘a friend and a foe’ for RTW after a TBI. These findings provide implications for both research and clinical practice.

### Clinical implications

Participants in this study described how distractions impacted their cognition as well as critical areas of executive functioning such as inhibition, self-monitoring, planning, organization, working memory, and emotional control. Thus, the influential role of distractions may exacerbate the challenges with providing individualized treatment plans for persons with TBI and RTW^40^. Clinicians should consider the farther-reaching impact of distractions on areas such as executive functioning and social communication in addition to attention and memory when developing strategies for RTW. Yet, the continued challenge for clinicians is obtaining accurate descriptions of distractibility in everyday life due to deficits in long term memory, insight, and awareness after a TBI^26^. However, emotional memory is often less impaired if not within normal limits^42,43^. Authors have identified that participants within this study provided rich descriptions of cognitive and executive dysfunction due to workplace distractions when asked to describe how distractibility made them *feel*. Therefore, rich descriptions may have been elicited from authors inquiring about the emotional aspects of distractibility. Authors are preparing a second manuscript on this data to further explore this interview approach and to develop an interview guide for clinicians to utilize when discussing everyday distractibility and RTW.

Participants endorsed the importance of the combination of emotional status, fatigue level, repeated exposure to distractions and work tasks to help them identify strategies for RTW. Participants utilized strategies such as alarms, instrumental music, reviewing open computer tabs, a digital calendar for notes, working with others, and monitoring non-verbal communication. Based on interview responses from this study, the use of external distraction (i.e., instrumental music) as a distraction masker is a new strategy that may support persons with TBI and RTW. These findings are similar to previous research in the areas of neuroplasticity, attention deficit hyperactivity disorder (ADHD), and autism identifying external distractions as a tool for cognition^44–49^. However, the translation of how to utilize distractions in clinical practice is unknown. Clinicians are encouraged to individualize treatment plans and strategies based on the combination of individual factors (i.e., fatigue, emotional control), target environment, tasks, and distractions. Additionally, clinicians are encouraged to include distractions that are individualized to patient’s environments and connected to functional tasks. By including distractions into clinical practice, clinicians can elicit everyday behavior, identify individualized strategies, and facilitate the generalization of clinic-based performance to everyday life.

### Research implications

Participants’ descriptions of how distractions impacted their work performance is the first step in the development of customized simulation programs for persons with TBI and rehabilitation. Future research should investigate and compare the impact of distractions in common environments such as the home, school, and workplace to distraction-free environments (i.e., clinical setting) to increase the sensitivity of cognitive assessments. Additionally, future research should compare behavioral and user experience outcomes between simulation programs and traditional therapeutic interventions. However, more research is required to identify how to include distractions in clinical tools to elicit cognitive behaviors that are parallel to everyday life. This research may help in the development of simulated intervention programs where persons with TBI can trial recommended strategies without the steep consequences (i.e., financial, social, emotional) that occur in workplace environments. Additionally, work-related training programs that include distractions may facilitate an increased tolerance for distraction therefore minimizing their impact for persons with TBI. Furthermore, research on task enhancing and task disrupting distractions may assist in the development of work accommodations. For example, semi-skilled employment positions in retail often have background music that includes content (i.e., words) which was reported to have a negative impact on work performance as compared to instrumental music. Lastly, future research should also investigate differences in distractibility based on certain TBI demographics such as injury severity, time since injury, differences pre and post TBI, baseline assessment scores, and work or academic status.

Participants endorsed the significant impact of internal distractions on task performance, social communication, organization, planning, and self-monitoring. Consistent with previous findings by Nochi^50^, Riley and Hagger^51^, participants were fearful of being viewed as incompetent in the workplace which created a chronic internal distraction. The perceived stigma about distractibility in the workplace created fear and anxiety which limited participants’ willingness to disclose their TBI and utilize accommodations. Therefore, participants were able to inconsistently conceal their distractibility which created a chain effect on their emotional control and task completion^52,53^. Future research and clinical tools such as assessments, questionnaires, and interventions may need to be designed to include the role of internal distraction in everyday cognition.

### Limitations

This study does have limitations. Interviews were completed via phone calls due to the COVID-19 global pandemic. Therefore, the quality of participant responses could have been influenced by external and internal distractions that were not controlled by the investigator. Authors utilized the term distraction to inquire about positive and negative effects on task performance. This could have potentially biased participant responses and future research could inquire on how the utilization of distraction terminology influences participant responses. The gender ratio of male to female was 1:6 which differs from reported ratios of brain injury of 2:1^54^. The participants severity levels ranged from mild, moderate, to severe. Therefore, the impact of distractions could have varied depending on participant’s severity levels and previous work experience. Additionally, the timeframe of how long participants had been working at their jobs differed. This may have inadvertently impacted how well-adjusted participants were to workplace distractions, thus making them less noticeable. This may be a direction for future research to explore the impact of distractions depending on time since injury, severity, and work experience. Many persons with TBI could not return to in person employment due to the COVID-19 pandemic and this may have impacted the recruitment of participants for the current study. This factor may have contributed to the type of employment participants were able to have and full or part-time employment status. Additionally, the goal of qualitative research is not to generalize to the entire target population, therefore, the results are not generalizable for all persons with TBI. However, the data from this study can be applied to future clinical research.

## Conclusion

Rich descriptions of the impact of workplace distractions are absent in the current TBI literature and limit the development of clinically relevant tools for persons wanting to RTW. This study described the experiences of seven individuals with TBI and how they perceived workplace to impact their productivity. The results from this study contribute to the small body of literature investigating the impact of distractions for persons with TBI in the workplace. Data from this study suggests that distractions may be utilized to both disrupt and enhance task performance in employment-based settings for persons with TBI. Additionally, our findings indicated that distractibility impacted several areas of employment that were unrelated to task timing and accuracy. If future studies include environmental distractions within simulation programs, a rich understanding of participant experiences with distractions is needed to ensure the implemented distractions are eliciting the desired behavioral outcomes. To improve rehabilitation outcomes, we suggest that including distractions should be individualized to patient’s environments and connected to functional tasks.

## Supplementary Information

Supplementary Information.

## Data Availability

The datasets used and/or analyzed during the current study available from the corresponding author on reasonable request.
