# Supplementary material for: Navigating the impact of workplace distractions for persons with TBI: a qualitative descriptive study
Source: Sci Rep. 2022 Sep 23;12:15881. doi: 10.1038/s41598-022-20083-0 (PMC9508104; doi:10.1038/s41598-022-20083-0)
Supplement: Supplementary file 1 — Supplementary Information. [file 41598_2022_20083_MOESM1_ESM.docx]

Interview Guide

**Grand Question:**

What are the lived experiences of individuals with TBI with workplace performance in the presence of distractions?

| **Primary Question** | **Sub-Question** | **Sub-Question** | **Sub-Question** |
| --- | --- | --- | --- |
| What jobs or volunteer activities have you done since your injury? | Can you describe the types of environments these were in? |  |  |
| Do you feel that your workplace environment affects your productivity? Does it help or hinder? | What are the aspects of work that are distracting? (Prompt- visual (items in your environment), or auditory (other co-workers, customers, radio, machines)) | How do these distractions affect you or your work specifically? | What is the most significant distraction? Why? |
| What tasks make you more or less distracted? (Prompt-tasks that require you to be in a busy environment, talking with multiple people at once, tasks that require mental math or problem solving) | Are there certain times of the day where your environment is less or more distracting? (Prompt-mornings, afternoons, before the place of business opens to the public) | What are the general consequences for being distracted at work? (Prompt-Individual, being late completing work tasks, errors on work tasks. Professional-write ups from boss, disagreements with co-workers) |  |
| What consequences have you personally experienced as a result of being distracted at work? |  |  |  |
| What distractions enhance or help with your work performance? (Prompt-salient background noise (i.e., music, white noise)) |  |  |  |
| How do you feel when you are distracted at work? (Prompt-feelings of frustration, embarrassment, helplessness, annoyance) |  |  |  |
| How do you realize if you are distracted at work? (Prompt-When you have made an error, a significant amount of time has passed, when someone re-directs you) |  |  |  |
| What support is required to manage your distractions in the workplace? (Prompt: Additional time for projects, weekly meetings with supervisor or boss) | Are there any environmental modifications you require to manage workplace distractions? If so what are they? (Prompt: headphones, working early or late when less people are around, clutter free desk) |  |  |
